# Rapid hydrothermal cooling above the axial melt lens at fast-spreading mid-ocean ridge

**DOI:** 10.1038/srep06342

**Published:** 2014-09-11

**Authors:** Chao Zhang, Juergen Koepke, Clemens Kirchner, Niko Götze, Harald Behrens

**Affiliations:** 1 Institut für Mineralogie, Leibniz Universität Hannover, 30167 Hannover, Germany

## Abstract

Axial melt lenses sandwiched between the lower oceanic crust and the sheeted dike sequences at fast-spreading mid-ocean ridges are assumed to be the major magma source of oceanic crust accretion. According to the widely discussed “gabbro glacier” model, the formation of the lower oceanic crust requires efficient cooling of the axial melt lens, leading to partial crystallization and crystal-melt mush subsiding down to lower crust. These processes are believed to be controlled by periodical magma replenishment and hydrothermal circulation above the melt lens. Here we quantify the cooling rate above melt lens using chemical zoning of plagioclase from hornfelsic recrystallized sheeted dikes drilled from the East Pacific at the Integrated Ocean Drilling Program Hole 1256D. We estimate the cooling rate using a forward modelling approach based on CaAl-NaSi interdiffusion in plagioclase. The results show that cooling from the peak thermal overprint at 1000–1050°C to 600°C are yielded within about 10–30 years as a result of hydrothermal circulation above melt lens during magma starvation. The estimated rapid hydrothermal cooling explains how the effective heat extraction from melt lens is achieved at fast-spreading mid-ocean ridges.

Fast-spreading mid-ocean ridges (MOR) are a major birthplace of the oceanic crust on Earth. The axial melt lens, being about 1–1.5 km below the sea floor as small as <100 m in thickness and <2 km in width^1^,^2^,^3^, plays a critical role in feeding both the upper crust by magma ejection and the lower crust by crystal foundering^4^,^5^,^6^,^7^,^8^. The formation of oceanic crust is coupled with strong heat loss associated with interaction between magmatic and hydrothermal processes within the ridges^3^,^9^, and results in volatile and element transfer between sea water and lithosphere. This process also promotes global geochemical cycling of many biosphere-sensitive components^10^. The cooling rate of oceanic crust above the melt lens is controlled by an interplay of magma replenishments and overlying hydrothermal circulation and exerts an important effect on magma assimilation, alteration of oceanic crust and related element mobilizations^9^,^11^,^12^. Previous studies proposed two different tectonic models, namely the “gabbro glacier” model^3^ and the “sheeted sill” model^13^, for explaining the accretion style at fast-spreading ridges. The “gabbro glacier” model depicts that magma crystallization takes place exclusively in the axial melt lens at the base of sheeted dikes, while the “sheeted sill” model predicts supplementary sill-shaped crystallization sites below the melt lens. These two endmember models have contrasting thermal structures^14^ and the cooling rates at comparative depths of the MOR crust should also be very different^15^. The schematic model in Fig. 1 gives a general cross-section image of the crust structure at fast-spreading ridges, accordant to the “gabbro glacier” model which is favored by many other studies (see recent review in ref. 16). In this work, we used the so-called “diffusion speedometry” for plagioclase from four granoblastic hornfels samples obtained through deep drilling into the fast-spreading oceanic crust formed at the East Pacific Rise (EPR) to estimate the cooling rate above the axial melt lens. The results are used to constrain the timescale of melt lens activity and to shed light on the crustal accretion style at fast-spreading ridges.

The studied samples were recrystallized sheeted dikes recovered by the Integrated Ocean Drilling Program (IODP) multi-leg campaign “Superfast Crust” at Site 1256 on the Cocos plate of the eastern equatorial Pacific (Fig. 2, see also Supplementary Information). The drilled oceanic crust was formed at the EPR about 15 Myr ago during a period of superfast spreading (full spreading rate: 210 mm/yr)^17^. Near the bottom of Hole 1256D, two gabbroic intrusions have been recovered at depths of 1400–1500 meters below sea floor (mbsf), which might indicate the roof of a crystallized melt lens^17^. The lowest ~60 m of the sheeted dikes above the gabbros were transformed to granoblastic hornfels through metamorphic overprint, exhibiting a gradual downward progression of the degree of recrystallization, and are typical of granoblastic plagioclase, clinopyroxene, orthopyroxene and Fe-Ti oxides assemblages (see Supplementary Information). Four of the hornfelses close to the gabbroic intrusions were investigated in this study for intra-plagioclase zoning. Petrological studies suggest that the granoblastic mineral assemblages were transformed from sheeted dikes which experienced hydrous alteration, following the recrystallization under granulite-facies conditions (refs. 18,19,20) as a consequence of heating by the underlying magma. This contact metamorphic horizon thus represents an interface between the hydrothermal circulation system in the upper crust and the magmatic regime of the melt reservoir, and might be accompanied by partial melting of former sheeted dikes involving breakdown of hydrous minerals^21^. Cooling in this interface zone from such high temperatures (ca. 1000°C, ref. 19) to low-temperature hydrothermal fluid-dominated conditions (<600°C, ref. 20) plays a fundamental role in extracting heat from the melt lens^3^,^15^, but the cooling rate is poorly constrained.

Several lines of evidence suggest periodic replenishment of magma into the melt lens (refs. 8,22,23), which would inevitably induce alternating hydrothermal alteration and thermal recrystallization in the overlying sheeted dikes. The root of the sheeted dikes undergoes peak thermal overprint at t_0_ when the melt lens reaches its highest position and the hydrothermal circulation is blocked therein (Fig. 3), while later suffers hydrothermal alteration at t_1_ when the melt lens retreats as magma replenishment is waning or even stops. It is generally accepted that there are multiple cycles of such processes^22^, and the thermal and hydrothermal overprints formed at an earlier time are largely erased by later cycles. Therefore, the current information represents the last on-ridge cycle which is then followed by off-ridge spreading (Fig. 3).

Thermal modelling of fast-spreading ridges for a static melt lens indicates that the off-ridge cooling rate at the roof of the melt lens decreases with distance from the ridge centre and the largest temperature gradient occurs at the vicinity of melt lens^15^. This implies, for a dynamic melt lens model, that heat loss within the recrystallized sheeted dike overlying a retreating melt lens should be very intense. Combining the results of thermal modelling^15^ and Ti-in-amphibole thermometry^19^,^24^, we consider that the temperature in the roof rocks above the axial melt lens should be about 600°C when the off-ridge regime starts (at t_1_ in Fig. 3). The off-ridge cooling then proceeds, from about 600°C to 100°C within a relatively short spreading distance from the ridge centre, being about 6 km for a half fast-spreading rate of 100 mm/yr (Fig. 3).

Cooling rates for the lower oceanic crust of fast-spreading MOR systems were previously calculated based on the Ca-in-olivine diffusion speedometry^25^. Since olivine is absent in the contact metamorphic roof rocks above the axial melt lens, the olivine speedometry cannot work for modelling the cooling rates in this case. However, magmatic plagioclase phenocrysts in the hornfels samples which survived the granoblastic overprint, probably as a result of the sluggish character of high-Ca plagioclase during high-temperature reactions (refs. 26,27), offer another opportunity for applying diffusion speedometry to constrain cooling rate. Many relics of plagioclase phenocrysts in hornfelses show an inherited igneous core and an overgrowth rim equilibrated with the hornfelsic matrix (Fig. 4, see also Supplementary Information and ref. 19). The cores throughout the recrystallized sheeted dikes show euhedral habits and high An contents (65–75%) similar to the primary plagioclase phenocrysts in fresh lavas and dikes^28^, clearly indicating a magmatic origin of these plagioclase cores. In contrast, the plagioclase rims have much lower An contents (45–55%) like the matrix plagioclases which have grown during the granoblastic overprint. The overgrowth rims usually contain abundant microgranular inclusions of plagioclase, pyroxene and Fe-Ti oxides, which are identical to the matrix in mineralogy. This texture indicates that the overgrowth rims of plagioclase phenocrysts are products of thermal overprint, similar as found in other hornfelsic rocks at the gabbro-dike transition sampled from modern ocean crusts or from ophiolites^18^,^29^. Comprehensive petrographic studies on the drilled samples from the IODP Hole 1256D revealed a coherent increasing degree of the thermal-overprint recrystallization with depth, from non at shallow sections through low-amphibolite facies to granulite facies (or two-pyroxene hornfelsic facies) at the dike/gabbro contact^19^. The hornfelsic samples investigated in this study were collected just above the uppermost gabbroic intrusion (see Supplementary Information) and thus should record the highest degree of thermal overprint.

For simplicity, we assume that each overgrowth rim has a homogenous composition and formed very fast at the peak temperature of thermal overprint (at t_0_ in Figs. 3–4). Analyses of magmatic cores of the plagioclase phenocrysts indicate two different cases: (1) the magmatic core is homogeneous and (2) a high-An rim surrounds the magmatic core. In the second case, the high-An rims are interpreted as being generated by an individual crystallization event induced by changing conditions, i.e., mixing with a more primitive or relatively water-rich melt^30^,^31^.

Subsequent to the peak thermal overprint in the root of the sheeted dikes, the temperature decreases as a combined result of magma starvation in melt lens and hydrothermal circulation. Amphibole crystallization at that stage is ubiquitous, occurring both as individual grains and overgrowth around clinopyroxene cores (Fig. 4), which indicates a wide temperature range of hydrothermal alteration from up to 1000°C to lower than 600°C^19^,^20^. Analyzed whole-rock δ^18^O values of sheeted dikes show gradual decrease with increasing depth towards the dike/gabbro transition zone^32^, also evidencing high-temperature (>600°C) hydrothermal alteration exclusively near the top of the melt lens. Having undergone on-ridge cooling and subsequent off-ridge cooling (Fig. 2), the initial sharp step in plagioclase An content between the magmatic core and granoblastic overgrowth would be broadened to some extent due to thermally induced intra-plagioclase diffusion (Fig. 4). In this study we compare the modelled diffusion profiles to the measured ones to reveal the timescales and cooling rates associated with the formation of the intra-plagioclase diffusion profiles. However, as stated previously and illustrated in Fig. 3, multiple cycles of thermal recrystallization and hydrothermal alteration should occur above the melt lens, which might result in complicated pulsing in An profiles at the core-rim boundary of plagioclase phenocrysts rather than the simple zoning depicted in Fig. 4. Because numerical modelling for such unconstrained pulsing is almost unrealistic, we obviate this problem by selecting plagioclase phenocrysts showing simple zoning but negligible pulsing and modelling the timescale for a single cooling process.

## Results

Constraining the peak temperature of the hornfelsic overgrowth is a key issue in calculating cooling rate. In this study, we applied the two-pyroxene thermometry^33^ to estimate the peak temperature of thermal overprint in the hornfelses. As shown in Fig. 5, three studied samples, 203R-1-10_14, 205R-1-10_14 and R12-B, indicate an identical upper limit temperature of about 1050°C, and this value is thus used as the peak temperature for modelling cooling rates of these three samples. One sample R12-S has an upper limit temperature of about 1010°C and a wider temperature range down to 850°C, which demonstrates that the peak temperature of granoblastic thermal overprint of R12-S is lower than the other three samples. These temperature estimates are consistent to those made for hornfelses in the dike/gabbro transition zone from other locations at EPR and from ophiolites^29^.

Taking into account the estimated peak temperatures, the boundary condition of an on-ridge regime and an assumption of linear cooling at hydrous conditions, we modelled the An% content profiles applying a forward finite-difference approach for the four granoblastic dike samples using CaAl-NaSi interdiffusion at hydrous conditions determined by Liu and Yund (1992)^34^. As shown in Fig. 6 (also Fig. S6–S9 in Supplementary Information), the modelled on-ridge cooling rates for CaAl-NaSi interdiffusion are about 30 ± 15°C/yr for the investigated four samples, corresponding to timescales of 10 to 30 years for the production of the CaAl-NaSi profiles. The small discrepancies from different measured profiles and estimated cooling rates might potentially result from several factors, such as the effect of crystallographic orientation on diffusion rate^35^, small-scale heterogeneous distribution of temperature and/or water activity, and non-linear temperature changing routes. However, further detailed exploration of the possible causes of the discrepancies from different profiles will not change the range of the modelled timescales and thus is out of the scope of this study. From an overall perspective, the estimates of cooling rate apply to both cases of the magmatic cores, i.e. the magmatic plagioclase cores with or without a high-An rim. It should be noted that the off-ridge cooling below 600°C does not modify the profiles significantly as indicated by our calculations, but low-temperature late-stage hydrothermal activities should have occurred as documented by alteration phase assemblages mainly involving quartz, chlorite, and etc, showing a major temperature range of 250–500°C^20^. The estimated on-ridge cooling timescales are consistent with inferred yearly to decadal variations in the melt lens geometry^36^ and the timescales of lava eruption^37^,^38^ and shallow hydrothermal discharge^39^ at fast-spreading MORs. This implies that hydrothermal cooling in the hornfelses at the roof of the melt lens and fluctuation of magma replenishment into the melt lens are coupled with each other in time.

Trace diffusion of Mg in plagioclase is another useful speedometry in constraining timescales of high-temperature geological processes^40^. Based on measured Mg concentration profiles, we modelled Mg diffusion in plagioclase which occurred simultaneously with CaAl-NaSi interdiffusion (see detailed modelling methods in Supplementary Information). The modelled Mg diffusion rates imply very low SiO_2_ activity in the hydrothermal fluids during the on-ridge cooling processes down to 600°C (Fig. 7), provided that the experimentally determined dependence of Mg diffusivity on SiO_2_ activity^41^ can be extrapolated to such low SiO_2_ activity. An important support for this hypothesis is given by a study on the saturation temperature of the fluid inclusions in quartz grains recovered from the IODP Hole 1256D^20^, which shows that all the quartz has crystallized from hydrothermal fluids at temperatures < 450°C and thus implies that the SiO_2_ activity should be very low at higher temperatures.

## Discussion

In order to examine the validity of the estimated cooling rate above the axial melt lens, we performed a simple heat balance calculation to compare the total heat released from the melt lens and that extracted from the overlying hornfelsic zone during cooling (see Methods for the procedure). The calculation assumes (i) that all the heat originated from cooling and partial crystallization of the melt lens is released into the overlying hornfelsic zone which acts as a conductive boundary layer, and (ii) that the timescales of replenishment and starvation of the melt lens is identical to each other. In order to balance the heat output of a melt lens at a fast-spreading MOR similar to the case of IODP Hole 1256D, the cooling rate above the melt lens is required to be around 30°C/yr (Fig. 8). This independent estimation from heat balance calculation is strikingly consistent with that from the modelling of intra-plagioclase CaAl-NaSi interdiffusion. We conclude that rapid hydrothermal cooling above melt lens is therefore essential for extracting heat during the crustal accretion at fast-spreading MORs. This is in complete accord with the dramatic decrease in cooling rate with depth in the lower crust estimated from Ca-in-olivine speedometry, which indicates that cooling in the plutonic lower crust is driven by hydrothermal circulation from top^25^.

**Figure 1:**
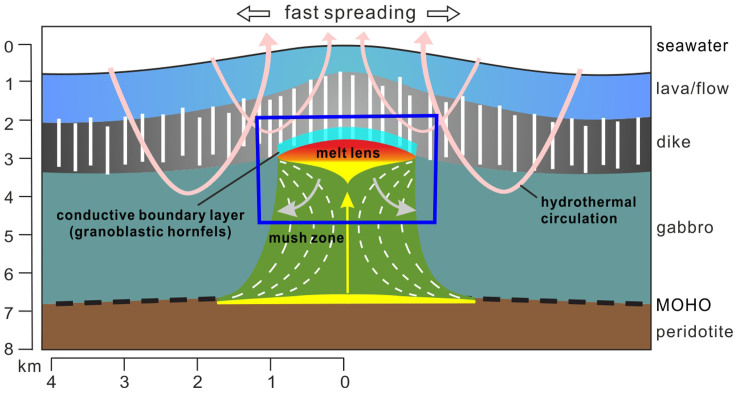
A schematic model of magmatic system at fast spreading mid-ocean ridges. The model is depicted based on combining ideas and evidences from numerous previous studies, mainly from refs. 2,3,6,7,43,44,45,46,47,48. The situation shown here is corresponding to a peak magma supply and shallowest position of melt lens, and in this situation, the conductive boundary layer atop of melt lens is nearly totally composed of granoblastic hornfelses (recrystallized sheeted dike). The whole axial magma chamber is composed of a melt zone at the top of upper mantle, a mush zone and an axial melt lens in the upper crust. The mush zone has a much higher fraction of crystals compared to the melt zone and axial melt lens, which are assumed to consist of pure liquid (e.g., ref. 1). The melt ascent zone in the lower crust (yellow arrow) denotes the path along which melts rise up, and it might be numerous small dikelets rather than a single tube as shown here. The white dashed curves in the mush zone are isotherms, and the grey solid curves indicate transport vectors of crystalline phases. A dynamic model for the evolution of melt lens defined by the blue rectangle is shown in Fig. 3.

The fast energy release above the melt lens might promote enhanced magma cooling and crystallization within the melt lens^42^ and is thus important in constraining the geological model for crustal accretion at fast-spreading MORs. The two commonly evoked tectonic models, namely the “gabbro glacier” model^3^ and the “sheeted sill” model^13^, interpret the thermal structure and heat extraction by contrasting ways. Recent estimation of magma crystallization depths at the fast-spreading EPR^5^ indicates that most crystallization carried out within the melt lens, although a minor part of crystallization has occurred prior to the magmas reached the melt lens, supporting a hybrid “gabbro glacier”-dominating model. Furthermore, the primitive layered gabbros recovered from Hess Deep^4^ and the strong mush subsidence recorded in the frozen axial magma chamber of the Oman ophiolite^6^ also support that a large proportion of magma crystallization occurred within the shallow melt lens and subsequently the crystals sank to form the mush zone (Fig. 1). The rapid cooling rates above the melt lens determined in this study, as well as the coupled timescale of cooling and melt lens fluctuation, indicate that effective heat extraction from the top of the melt lens can be achieved through overlying hydrothermal circulation within the overlying sheeted dikes (Fig. 8). Therefore, our data support a model suggesting that hydrothermal circulation above the melt lens plays a dominant role of heat extraction for solidifying the lower crustal at fast-spreading MORs, in favor of the “gabbro glacier” model (for details see review in ref. 16). The melt lens might wax and wane in a yearly to decadal timescale, which can explain the observed variations in melt lens geometry^36^, lava eruptions^37^,^38^ and shallow hydrothermal discharge^39^ at fast-spreading MORs.

## Methods

### Electron microprobe analysis

Mineral spot compositions and intra-plagioclase profiles were measured using a Cameca SX100 electron microprobe analyzer equipped with 5 spectrometers and an operation system “Peak Sight”. Spot analyses of all mineral phases were performed with an acceleration potential of 15 KeV and a beam current of 15 nA. For analyzing Na, Si, Al and Ca of intra-plagioclase profiles, firstly a normal setting of 15 KeV and 15 nA has been used. In order to improve analytical spatial resolution for very short diffusion distances (5 ~ 15 μm) within plagioclase, that is, to diminish excitation volume of analytical spot, we used a modified setting of 8 KeV and 10 nA for measuring most intra-plagioclase profiles for Na, Si, Al and Ca, but trace Mg, K, Fe and Ti were not analyzed in this case. The spatial resolution of this setting has been tested to be able to fulfill the requirement of the measurement (see Supplementary Information). For analyzing the MgO content of the intra-plagioclase profiles, 15 KeV and 40 nA have been used in order to improve the detection limit, which can analyze Mg > 55 ppm with an uncertainty about 70 ppm.

### Diffusion modelling

Initial step-like transitions in An content at the interface were assumed for forward numerical modelling applying a finite-difference approach, similar to the method used in ref. 40. Both on-ridge and off-ridge cooling processes were considered in diffusion modelling by superimposing the latter one on the former output. For both processes simplified linear cooling rates were assumed, which might slightly overestimate the timescale of on-ridge cooling^22^ and underestimate the timescale of off-ridge cooling^15^. The diffusivities for CaAl-NaSi interdiffusion (D_CaAl-NaSi_) and for Mg trace diffusion (D_Mg_) at hydrous conditions were taken from ref. 34 and ref. 41 respectively, and the temperature-dependent equation can be expressed as 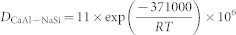 and 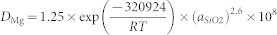. In the equations, *R* is ideal gas constant, *T* is temperature in Kelvin, and *a*_SiO2_ is silica activity in the system, and the unit of diffusivity is μm^2^s^−1^. We chose the diffusivity data based on careful examination of the available diffusion experiments (see details in Supplementary Information).

### Heat balance calculation

**Figure 3:**
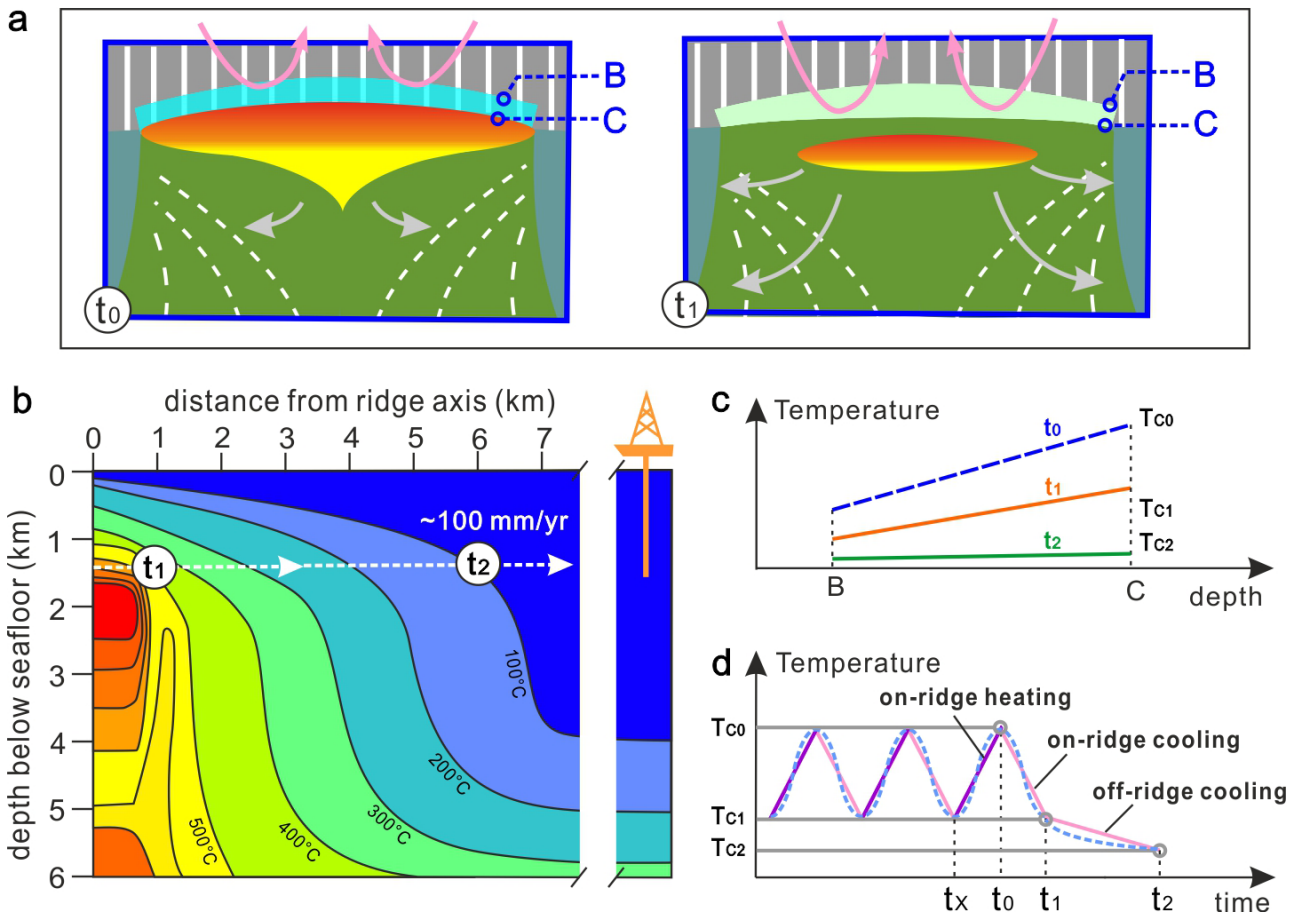
Geological model for estimating cooling rates of the hornfelsic rocks above melt lens at a fast-spreading ridge. (a): A dynamic perspective of the MOR structure changing from time t_0_ (melt lens is at the shallowest position within the crust resulting in peak thermal overprint of the overlying sheeted dikes) to time t_1_ (melt lens is at the lowest position and the thermal-overprinted sheeted dikes are hydrothermally cooled). Note that crystallization in the melt lens and subsequent crystal sinking are stronger at t_1_ than at t_0_. Positions B and C denote, respectively, the top and the base of thermally overprinted sheeted dikes. (b): Static thermal structure near MOR (after ref. 15). The IODP Site 1256 is about 1200 km away (15 Myr old) from the spreading centre. The spreading rate on the panel (100 mm/yr) is half spreading rate. (c): temperature variations with depth at the reference positions of B and C. (d): temperature variation at the base of sheeted dikes (position C). Subsequent to the latest on-ridge heating by the melt lens to the latest peak thermal overprint (from t_X_ to t_0_), there were two cooling stages: on-ridge cooling from t_0_ to t_1_, and off-ridge cooling from t_1_ to t_2_. The temperature variation from t_2_ to present time is minimal and ignored. The average off-ridge cooling rate from time t_1_ to t_2_ is estimated to be about 0.01°C/yr for an average half spreading rate of 100 mm/yr. The dashed curve in d shows another hypothetical temperature pattern if considering a sinusoidal melt lens fluctuation^22^ and decreasing off-ridge cooling rate outwards^15^.

**Figure 5:**
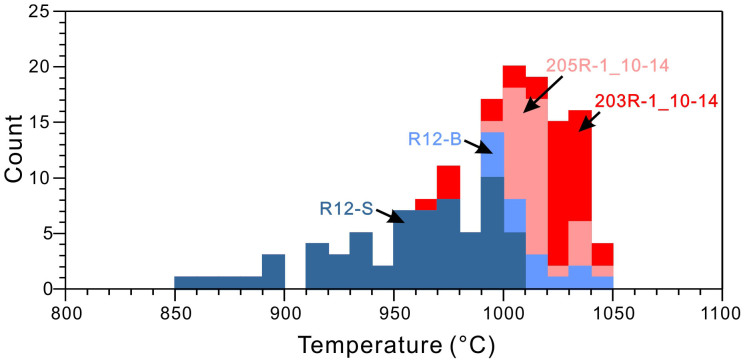
Temperature estimation of the granoblastic hornfelses. Temperatures are estimated by two-pyroxene thermometry (ref. 33), derived by analyzing coexisting clinopyroxene and orthopyroxene grains adjacent to relic plagioclase phenocrysts. Note that although sample R12-S has a longer temperature span than other three samples, its upper limit temperature is lower.

**Figure 6:**
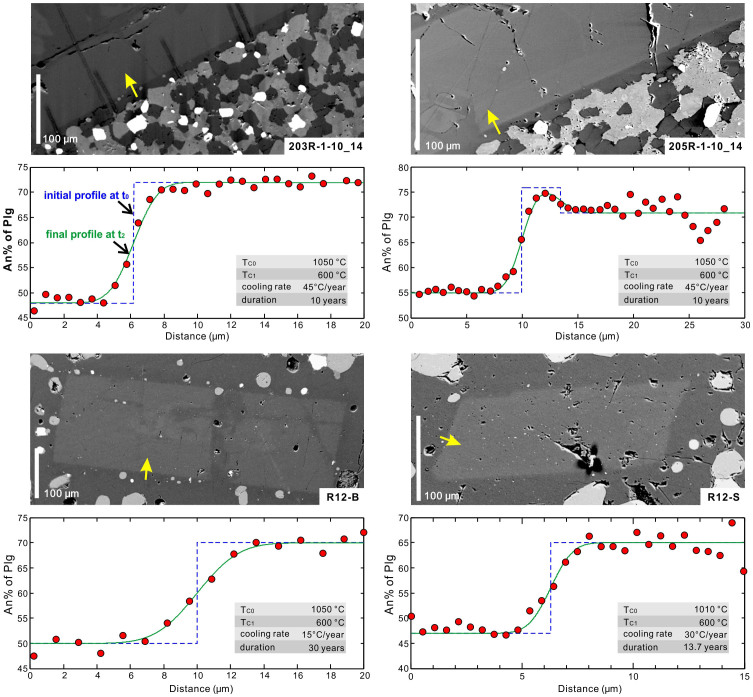
Images, concentration-distance profiles and modeled profiles for intra-plagioclase CaAl-NaSi couple interdiffusion. The measured An% contents along boundary-cutting profiles (denoted by arrows on back-scattered electron images) are shown as red dots. The best-fit final profiles at time t_2_ (green curves) were calculated involving two cooling steps (see Fig. 3) from the assumed initial profiles at time t_0_ (blue dashed curves). T_C0_ at t_0_ is estimated based on two-pyroxene thermometer (see Fig. 5). See more profiles and modelling results in Supplementary Information.

**Figure 7:**
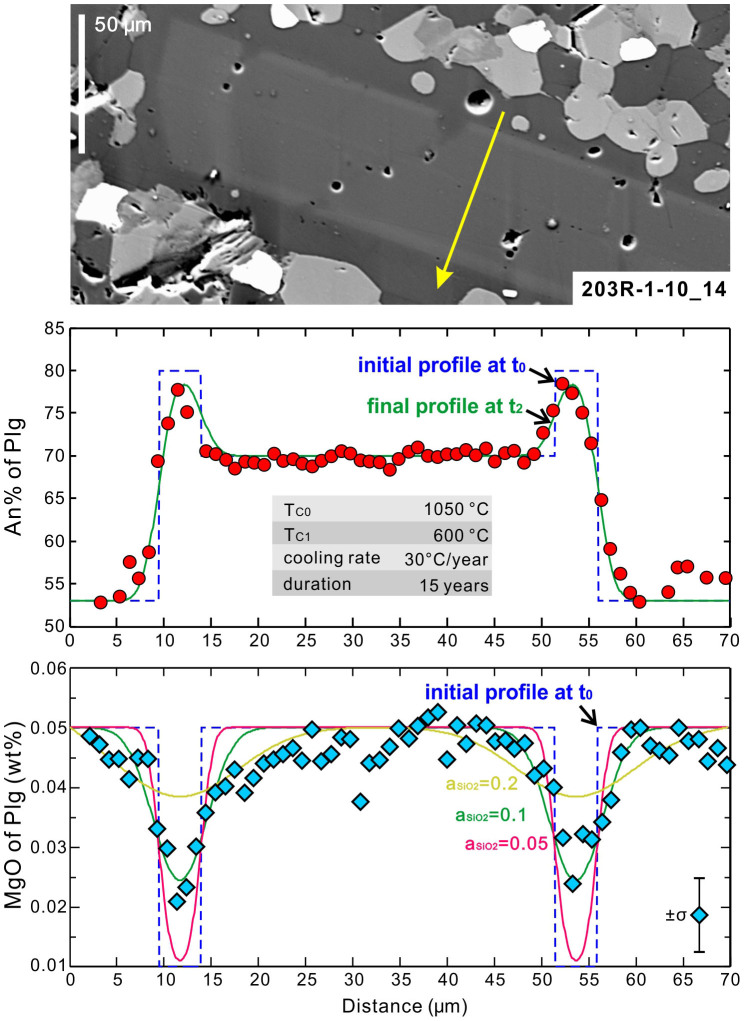
Image, concentration-distance profile and modeled profile for intra-plagioclase CaAl-NaSi couple interdiffusion and Mg trace diffusion. Sample 203R-1-10_14. The measured profile is denoted by yellow arrow on the back-scattered electron image. The assumed initial profiles at time t_0_ are denoted by blue dashed curves for both An% and MgO wt%. Modelled profiles for Mg trace diffusion using three different values of SiO_2_ activity (a_SiO2_) are denoted by curves in different color.

**Figure 8:**
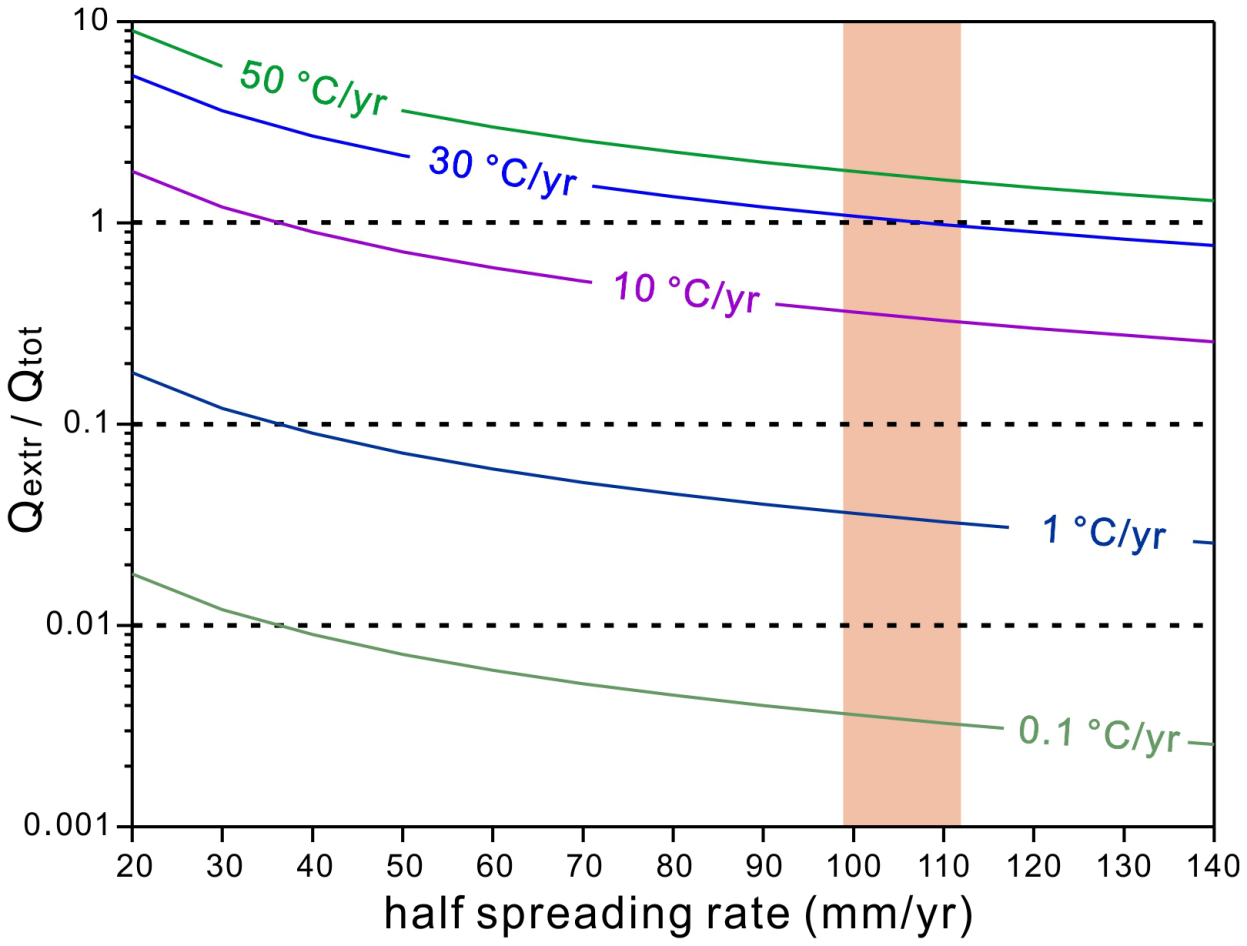
Heat balance calculation showing the correlation between ridge spreading rate and cooling rate above melt lens. Q_tot_ is total heat originated from melt lens by cooling and partial crystallization; Q_extr_ is heat extracted out of the recrystallized sheeted dikes via hydrothermal circulation after retreat of melt lens. Heat flux is balanced when Q_extr_/Q_tot_ = 1, which may be achieved by hydrothermal cooling above melt with a cooling rate of about 30°C/yr for a fast-spreading ridge with an average half spreading rate of about 100–110 mm/yr.

The calculation method used here follows principally that of ref. 43, and the used physical quantities are listed in Table 1. Within an episode of melt lens fluctuation (β, from t_X_ to t_1_, Fig. 3), the total heat released from half melt lens originates from magma cooling to the solidus and from latent heat of crystallization, which can be expressed as: Q_tot_ = uβρ_m_hC_m_ΔT_m_ + uβρ_g_hL. During the on-ridge cooling process, the heat extracted from the hornfelsic zone by hydrothermal circulation can be expressed for half-ridge width as Q_extr_ = aρ_d_kC_d_ΔT_d_. Assuming constant cooling rate r, the average temperature decrease in the hornfelsic zone during the cooling process (θ, from t_1_ to t_0_, Fig. 3) can be calculated by ΔT_d_ = 0.5θR. For an episode of melt lens fluctuation, we assume that the timescales of melt lens waxing and waning are identical to each other. Therefore, we have β = 2θ. Finally we calculate the ratio of Q_extr_/Q_tot_ as a function of the cooling rate above the melt lens and the half spreading rate, and the results are illustrated in Fig. 8.

### Error analysis

Errors of the cooling rates obtained in this study may come from several potential sources, mainly including: (1) uncertainty due to the spatial resolution of the electron beam, (2) nonlinear cooling path, (3) uncertainty in temperature estimation for peak thermal overprint, and (4) uncertainty in experimentally determined diffusion coefficients. Evaluations on these potential factors indicate that our modelled cooling rates using the assumptions are reasonable within a relative error of a factor of 2 (see details in Supplementary Information).

## Author Contributions

J.K. and H.B. initiated the research proposal. J.K. collected the samples. C.Z., C.K. and N.G. performed microprobe analysis. C.Z. and H.B. performed modelling. C.Z. and J.K. wrote the paper.

## Supplementary Material

Supplementary Information Supplementary Information

## Figures and Tables

**Figure 2:**
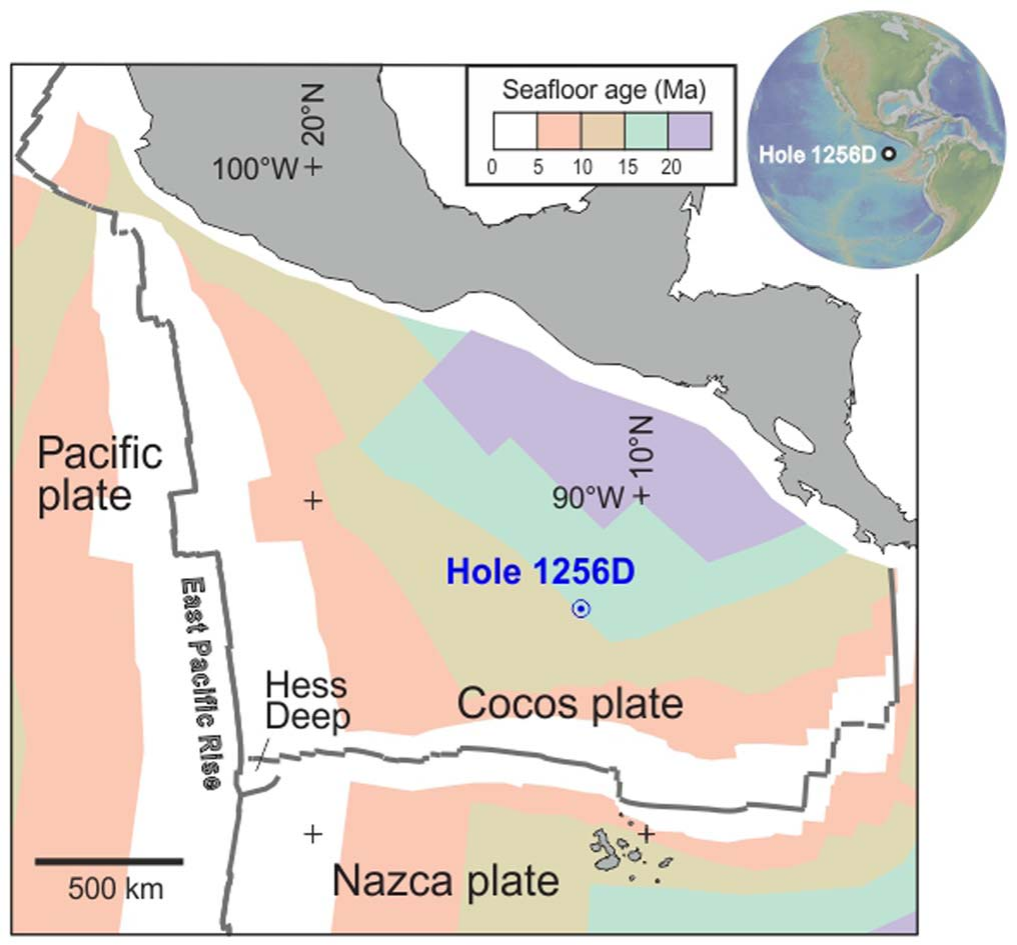
Tectonic position of the IODP Hole 1256D. Modified after ref. 17 with permission.

**Figure 4:**
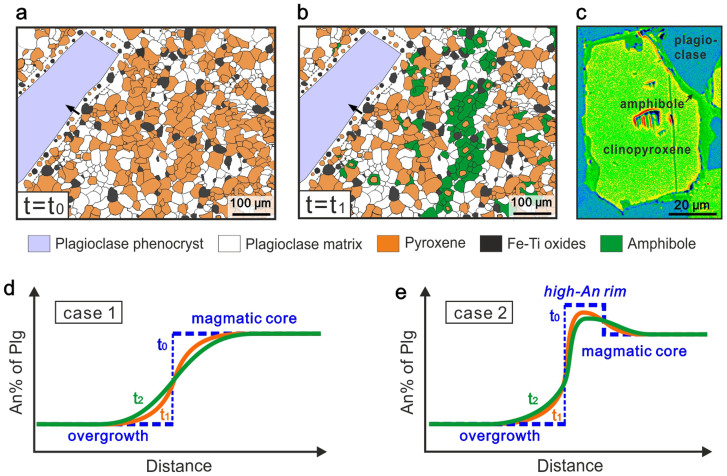
Petrologic model for intra-plagioclase diffusion modelling. (a): Sketch of the granoblastic texture in metamorphosed sheeted dikes surrounding a relic plagioclase phenocryst. The overgrowth on inherited primary plagioclase phenocryst formed at t_0_ as a result of thermal overprint. (b): Penetrative amphibole (mainly as alteration product of pyroxene) is formed between t_0_ and t_1_ as a result of hydrothermal overprint indicating a hydrous condition during cooling. (c): A pseudocolor BSE image shows that amphibole often encloses residual clinopyroxene cores, indicating cooling under hydrous conditions (sample R12-B). (d–e): Plagioclase anorthite content (An%) profiles across the core-rim boundary (profile position denoted as black arrow in (a) and (b)) at different times show the effect of intra-crystal diffusion on modifying the core-rim chemical contrasts. Case 1 (d): magmatic core of plagioclase has a homogeneous composition. Case 2 (e): magmatic core of plagioclase has a high-An rim. See Fig. 3 for geological meanings of t_0_, t_1_ and t_2_.

**Table 1:** Physical parameters used in heat balance calculation

Physical meaning	Parameter	Value	Unit
Total heat released from melt lens	Q_tot_		J
Heat extracted by hydrothermal flux	Q_extr_		J
Density of basaltic magma	ρ_m_	2800	kg m^−3^
Density of gabbroic lower crust	ρ_g_	3000	kg m^−3^
Density of recrystallized sheeted dike	ρ_d_	2700	kg m^−3^
Depth of gabbroic lower crust	h	5000	m
Temperature interval between liquidus and solidus	ΔT_m_	200	°C
Average temperature decrease in hornfels	ΔT_d_		°C
Depth of recrystallized sheeted dike	k	50	m
An episodic duration of melt lens fluctuation	β		yr
Duration of on-ridge cooling	θ		yr
Specific heat of basaltic magma	C_m_	1100	J kg K^−1^
Specific heat of recrystallized sheeted dike	C_d_	800	J kg K^−1^
Latent heat of crystallization of gabbro	L	500000	J kg^−1^
Half width of melt lens	a	1000	m
Cooling rate at the root of sheeted dike	R		°C yr^−1^
Half spreading rate	u		m yr^−1^
