# Supplementary material for: Rapid hydrothermal cooling above the axial melt lens at fast-spreading mid-ocean ridge
Source: Sci Rep. 2014 Sep 11;4:6342. doi: 10.1038/srep06342 (PMC4160713; doi:10.1038/srep06342)
Supplement: Supplementary Information [file srep06342-s1.pdf]

Supplementary Information

Rapid hydrothermal cooling above the axial melt lens at fast-spreading mid-ocean ridge

Chao Zhang, Juergen Koepke, Clemens Kirchner, Niko Götze and Harald Behrens

Geological background:

Integrated Ocean Drilling Program (IODP) Hole 1256D was initially drilled by Ocean Drilling Program (ODP) Leg 206 and then continued by IODP Expeditions 309, 312 and 335. IODP Hole 1256 is located in the 15-Ma old ocean crust of the Cocos plate, which was formed during a superfast spreading period at the East Pacific Rise (full spreading rate ~210 mm/year, Fig. 2)[1]. This drilling now yield a total depth of 1521.6 meters below seafloor (mbsf), which penetrated the whole upper oceanic crust composed of extrusive lava (~800 m thick), sheeted dike complex (~350 m thick) and finally into the uppermost gabbros (Fig. S1)[2,3], believed as part of the uppermost frozen axial magma chamber (AMC) system, were first encountered at 1406.6 mbsf. This shallow depth is consistent with seismic surveys on MORs and the prediction that the depth of low-velocity zone is in negative correlation with spreading rate[4].

The drilled gabbros consisting of two individual intrusions penetrated at depths between 1406 and 1495 mbsf (Fig. S1), which are demonstrated to have undergone crystallization at locus and hybridization between evolved and relative primitive magmas[2,5]. These two gabbro bodies form intrusions into the so-called "granoblastic dikes" (Fig. S1)[2,6], which are interpreted as former sheeted dikes, recrystallized under metamorphic conditions up to the granulite facies to hornblende and pyroxene hornfelses due to the contact metamorphism induced by the thermal imprint of a high-position AMC[6]. Furthermore, low degree of partial melting might have occurred upon hydrothermally altered sheeted dikes during contact metamorphism, extracting silicic melts and leaving the granoblastic dikes as residues[7,8]. Similar phenomena in such a dike-gabbro transition zone have also been observed in ophiolites[7,9-11] and thus are considered as a diagnostic feature of conductive boundary layer (CBL) which separates the overlying hydrothermal circulation system and the underlying basaltic magma system. Previous studies have shown that there is an apparent down-hole enhancement of metamorphic grade of the granoblastic dikes above the shallower gabbro intrusion[2,6], and in this study we focus on several selected samples of recrystallized sheeted rocks recovered at depths close to the gabbroic intrusions (at about 1400-1500 mbsf, Fig. S1), aiming to best reflect the cooling process as the AMC retreats which has been acting as the heat source for thermal overgrowth of adjacent sheeted dikes.

Sample descriptions:

Four representative pyroxene hornfels samples, 203R-1-10_14 (IODP sample 312-1256D-203R-1, 10-14 cm), 205R-1-10_14 (IODP sample 312-1256D-205R-1, 10-14 cm), R12-B (IODP sample 335-U1256D-RUN12-RCJB-Rock B) and R12-S (IODP sample 335-U1256D-RUN12-RCJB-Rock S) are the objects of this

study. These samples were selected from ~60 samples available showing the best condition for the aim of this study (in terms of presence of suited plagioclase phenocrysts, secondary hydrothermal alteration). 203R-1-10_14 and 205R-1-10_14 were collected during Expedition 312, closely above the first gabbro intrusion. R12-B and R12-D were collected during Expedition 335 from junk baskets, and their exact positions cannot be determined but are most likely beneath the second gabbro intrusion (ref. 3; Fig. S2). Their groundmasses typically form very fine-grained mosaic granoblastic textures consisting of plagioclase (Plg), clinopyroxene (Cpx), orthopyroxene (Opx) and Fe-Ti-oxides (Ox) [mostly magnetite (Mgt) and ilmenite (Ilm)], which indicates a high degree of hornfelsic-granulite facies contact metamorphism[6,12]; however, there are imperceptible differences of these samples which might reflect differences in primary magmatic texture and/or thermal imprint and also possibly hydrothermal alteration during cooling. Amphibole (Amp) occurs, besides in hydrothermal veins, in “wet” domains, and the boundary between amphibole-free “dry” and amphibole-bearing “wet” domains are diffuse. The readers are referred to ref. 6 for a detailed petrographical description of a variety of hornfels samples collected from Hole 1256D.

Sample 203R-1-10_14 is composed of several different petrological domains with sharp boundaries, including granoblastic domains, amphibole veins and amphibole-rich dioritic patches. The granoblastic domain consists of fine-grained granoblastic matrix (grainsize ca. 10-20 μm) and minor euhedral Plg phenocrysts (Fig. S2a), and the latter are believed to be inherited from original sheeted dikes. The rim of Plg phenocryst encloses numerous microgranular inclusions of Plg, Cpx, Opx and Ox (Fig. S2b), and this is an outstanding feature of thermal overgrowth observed in all granoblastic dikes (also see below). Within the granoblastic domain, two principal types of hydrothermal imprint exist: one type is characterized by sporadic occurrence of amphibole which altered former Cpx and coexists with Ox (Fig. S3a), indicating penetrative hydrothermal fluids at a high temperature; the other type occurs as chlorite veinlets which cut the granoblastic domain sharply (Fig. S3b) suggesting a vein-controlled hydrothermal activity at a low temperature.

Sample 205R-1-10_14 contains several petrological domains of distinctive textures and mineral assemblages within a thin-section scale, including dry and wet granoblastic domains, low-temperature and high-temperature amphibole-rich veins[6]. Here we focus on the dry granoblastic domain, which is composed of a very fine grained matrix (grainsize ca. 10-15 μm) and minor euhedral Plg phenocrysts (<1 %). The fine-grained matrix consists mainly of granular Cpx, Opx, Ox and subhedral Plg which form the framework of the rock (Fig. S2c). Fractures and fissures in Plg phenocrysts contain a similar mineral assemblage as in the matrix but with a finer grainsize (Fig. S2d). There are abundant micrometer-sized inclusions in matrix minerals (Fig. S4a), and the mineral assemblages of these inclusions are similar to that of the matrix. No apparent orientation of minerals can be observed. This sample is of texture type 8 for granoblastic overprint defined by Koepke et al. (2008)[6].

Sample R12-B is composed of granoblastic matrix (>95 %), including granular Cpx, Opx, Ox and Plg as (Fig. S2c) and minor Plg phenocrysts (<1 %). The grainsize of matrix minerals is primarily within 30-60 µm, apparently larger than those in sample 205R-1-10_14. At some locations, Opx grains form linear arrangement spaced by interstitial Plg. Abundant micrometer-sized inclusions are observable within the matrix grains (Fig. S4b). The Plg phenocrysts show a distinct size (mostly >1 mm in length) from the matrix, and exhibit inherited magmatic euhedral core and metamorphic overgrowth, and the latter typically interfinger with matrix Plg and enclose crowded granoblastic grains which are compositionally the same as the main part of matrix but relatively smaller in size (Fig. S2f). The petrographical characteristic of R12-B indicates that the recrystallization degree should be equivalent to or even higher than texture type 8 of the rank suggested by Koepke et al. (2008)[6], because precursor magmatic textures can hardly be recognized, which infers a very high degree of recrystallization induced by contact metamorphism.

Sample R12-S exhibits a typical high-degree granoblastic overprint (texture type 8) as shown by granular Plg, Cpx, Opx and Ox and interstitial Plg in the matrix, which occasionally encloses Plg phenocrysts (Fig. S2g). Amp, as a secondary phase, is in this sample widely absent. The Plg phenocrysts usually show sharp boundaries between the core and the overgrowth as being seen under cross-polarized light (Fig. S2h). The overgrowth part encloses microgranular grains of Cpx, Opx and Ox which are not observable in the core, indicating that the core (believed as inherited remnant of igneous phenocrysts) has not been influenced by post-magmatic thermal overprint or hydrothermal alteration.

Test of spatial resolution of microprobe analysis:

In order to test the spatial resolution of the microprobe for the two conditions used, we applied these settings to a special sample where pairs of plagioclases with different compositions were produced experimentally by a partial melting reaction. The partial melting of plagioclase single crystals under dry conditions produces for relatively short run durations (e.g. 24 hours) zoned plagioclase complexes with sharp boundaries between the composition of the starting plagioclase and the new equilibrium plagioclase precipitated from the melt. For details of the reaction see Johannes et al. (1994)[14]. This melting reaction is structure-controlled, and produces plate-like shaped melt pools and new An-enriched plagioclase with a preferred orientation parallel to the main cleavage, (001). Thus, when orientating sections of such partial molten plagioclase single crystals perpendicular to the a-axis or parallel to (001), the arrangement of the platy plagioclase pairs is perpendicular to the surface, providing numerous concentration steps with sufficient sharp contacts between two different plagioclase compositions, thus representing numerous opportunities for testing the local resolution of the microprobe for analysing plagioclase with a similar composition as in our natural samples. Diffusion, which might broaden the concentration step between the starting plagioclase and the newly formed plagioclase, can be ignored

due to the extreme low diffusivity of NaSi-CaAl interdiffusion in plagioclase under dry conditions in combination with relatively short run durations.

We reproduced the plagioclase single crystal melting experiment of Johannes et al. (1994)[13] using the same starting material (plagioclase from Lake County with an An content of 68 mol%) and the same experimental conditions (pressure: 1 atm, temperature: 1420°C, dry conditions, run duration 24 h). Back-scattered electron images reveal experimental results with the same phase situation as shown in Fig. 2B of Johannes et al. (1994). After the experiment, the crystals were cut parallel to (001) and concentration profiles across the contact of pairs of plagioclase starting material (An 68) and newly formed plagioclase (An 80) were analyzed applying the same analytical conditions as used for the natural samples. As expected, the obtained profiles did not show an ideal concentrations step, but a measurable distance between the two plagioclase concentrations corresponding to the local resolution of the electron beam. By measuring this distance we estimated for the 15 kV analytical setting a local resolution of 1.44 ± 0.14 µm and for the 8 kV setting a local resolution of 1.17 ± 0.20 µm.

Method of diffusion modelling:

Diffusion modelling was carried out using a forward modelling approach with finite difference method similar to the that used in Costa et al. (2003)[14] and Kahl et al. (2011)[15]. As illustrated in Fig. 3, we aim to model the cooling rate of the granoblastic hornfelses from the peak contact metamorphism to the condition where the off-axis regime starts, that is, from temperature T_{C0} at t_0 to T_{C1} at t_1 with a constant cooling rate of CR_1. This cooling process results from a retreat of the underlying melt lens and the onset of hydrothermal circulation. T_{C0} is estimated from two-pyroxene thermometry, because recrystallization of clinopyroxene, orthopyroxene and plagioclase are simultaneous as a result of being heated by the melt lens. For different samples, T_{C0} might be different dependent on the heat. T_{C1} is estimated from Ti-in-amphibole thermometry, because amphibole in the diagnostic phase resulted from hydrothermal alteration during on-ridge cooling. For samples from the lowermost sheeted dikes above gabbros at around 1400-1500 mbsf, we assume that the lower limit temperature of on-ridge cooling at this depth is 600 °C as determined from Ti-in-amphibole thermometry[6]. The off-ridge cooling process starting from T_{C1} at t_1 is assumed to be independent of on-ridge cooling process, and the corresponding cooling rate can be calculated from geothermal models[16] as 0.01 °C/year from 600 °C at ridge border to 100 °C (T_{C2}) at t_2 at about 6 km from ridge axis.

We use the finite difference equation for diffusion along a plane sheet[17], which can be written as

$$C_{t+dt}^{x} = C_t^x + D\frac{dt}{(dx)^2}(C_t^{x-dx} - 2C_t^x + C_t^{x+dx})$$

In the calculation, C is the quantity of anorthite content in this case, dx is set as 0.01 µm and dt is set by the computation of

$$dt = 0.1 \times (dx)^2/D$$

In the iteration calculation using MATLAB©, a first path from T_{C0} to T_{C1} and a second path from T_{C1} to T_{C2} with a known cooling rate (0.01 °C/year in this case) are combined consequently to search the best-fit cooling rate of the first path.

CaAl-NaSi interdiffusion: In order to model CaAl-NaSi interdiffusion in plagioclase for this case study, we examined firstly the available diffusion data in literature. There have been several experimental studies on determining the diffusivity of CaAl-NaSi interdiffusion in plagioclase at both dry and hydrous conditions, and a recent review can be found in Cherniak (2010)[18]. As shown in Fig. S5, comparison of experimental data from different researchers in principle indicates that (1) CaAl-NaSi interdiffusion in plagioclase at hydrous conditions is faster than at dry conditions by two log units or more and (2) interdiffusion in low-An plagioclase is significantly faster than that in high-An plagioclase. Although there remain some uncertainties in experimental protocols and assumptions made in evaluation of experimental data (see review in ref. [18]), previous diffusion experiments have demonstrated that the diffusion rate is strongly dependent on H_2O activity and plagioclase An content. Concerning this case study, the interdiffusion of CaAl-NaSi between magmatic core and overgrowth rim during hydrothermal cooling (Fig. 4) is believed to have occurred within An_{50}-An_{70} under hydrous conditions. Therefore, by evaluating the available data in literature, we consider that the situation in this case study is best represented by the study of Liu and Yund (1992)[19] on homogenization of An_{70}-An_{90} lamellas under hydrous conditions. As shown in Fig. S5, by extrapolating from lower-temperature data and omitting the inconsistent experimental data at 1000 °C (ref. [19]), we model CaAl-NaSi interdiffusion using the following equation:

$$D_{\mathrm{CaAl-NaSi}} = 11 \times \exp\left(\frac{-371000}{RT}\right) \times 10^{6}$$

in which D is diffusion coefficient in $\mu m^2 s^{-1}$, R is ideal gas constant, T is temperature in Kelvin. The best-fit cooling rates applying the above equation of diffusivity for the on-ridge cooling path are presented in Figs. S6-S9, and the geological implications are discussed in the main text. The potential uncertainty derived from diffusion coefficients is discussed in detail in “Error analysis” below.

Mg diffusion: There are several independent experimental studies on Mg diffusion in plagioclase, which are summarized in Fig. S10. Generally, the experimental data of LaTourrette and Wasserburg (1998)[20] and Van Orman (2014)[21] are in agreement with each other. One important finding was that An content of plagioclase exerts a strong negative correlation to Mg diffusivity[21]. In addition, Faak et al. (2013)[22] observed a strong increase of Mg diffusivity with silica activity (a_{SiO2}, 0.55-1) in their diffusion experiments with intermediate plagioclase compositions. It was demonstrated by Van Orman (2014)[21] that quartz was present in all of their experimental products, indicating silica saturation during experimental runs (a_{SiO2} = 1). The effect of silica on Mg diffusion can be explained by the formation of vacancies on cation sites, i.e. substitution of Al^{3+} plus $(Na, K)^{+}$ by Si^{4+} plus vacancy[23], and provides evidence that migration of Mg in plagioclase is controlled by a vacancy mechanism. Due to the unknown silica

activity in the granoblastic hornfelses, we cannot use the Mg profiles in the plagioclase for estimating cooling rates, but may use the equation of Faak et al. (2013)[22] to shed a light on the silica activity which was prevailing during the cooling process, when using the calculated cooling rates derived from NaSi-CaAl interdiffusion. The equation proposed by Faak et al. (2013)[22] is

$$D_{\mathrm{Mg}} = 1.25 \times \exp\left(\frac{-320924}{RT}\right) \times (a_{SiO2})^{2.6} \times 10^{8}$$

where *D* is diffusion coefficient in $\mu m^2 s^{-1}$, *R* is ideal gas constant, *T* is temperature in Kelvin, and a_{SiO2} is silica activity in the system. As shown in Fig. 7 and Fig. S11, the MgO zoning patterns of sample 203R-1-10_14 can be modeled with a_{SiO2} being about 0.05-0.2 to match the cooling rate derived from CaAl-NaSi interdiffusion. In contrary, the Mg diffusion coefficients in plagioclase determined by LaTourrette and Wasserburg (1998)[20] or Van Orman et al. (2014)[21] are inconsistent with the measured MgO profiles and the cooling rate derived from the An profiles, i.e., nearly flat MgO profiles would be expected. The estimated a_{SiO2} is out of the range of a_{SiO2} of 0.55 – 1 covered by the experimental conditions of Faak et al. (2013)[22], but support for very low silica activity is given by the study of Alt et al. (2010)[12] on the same drilling core (IODP Hole 1256D), which demonstrates that quartz in the root of granoblastic dikes have been only formed from hydrothermal fluids at very low temperatures (<430 °C) and silica activity should be very low at temperatures above 600 °C.

Error analysis:

Errors of the cooling rates obtained in this study may come from several potential sources, mainly including: (1) uncertainty due to the spatial resolution of the electron beam, (2) nonlinear cooling path, (3) uncertainty in experimentally determined diffusion coefficients, (4) uncertainty in temperature estimation for peak thermal overprint,. We discuss them briefly below.

(1) As stated above, the spatial resolution using the 8 kV setting is 1.17 ± 0.20 µm, and the resultant overestimation in length is less than 20% for 5-15 µm long diffusion. According to the general relation between diffusion length (x) and time (t), i.e., $x \approx (Dt)^{0.5}$, the resultant overestimation in diffusion time is estimated within 35%. Therefore, “true” profiles (without error in spatial resolution) might yield larger cooling rates by less than 35% than our estimates modelled from measured profiles, which means that our estimated cooing rates are minimum values considering spatial resolution errors.

(2) In the modelling procedure of this study, we assume a linear cooling path from peak thermal overprint (t_0 in Fig. 3) to the condition at ridge border (t_1 in Fig. 3), thus use a constant cooling rate for this process. However, in a natural case, the cooling rate might vary with time. For example, in the study of Rannou et al. (2006)[24] sinusoidal models of magma replenishment at mid-ocean ridges are used to explore the magma supply rate. It is expectable that if a sinusoidal model is used instead of linear model in this study, the obtained total cooling time scales should be slightly shorter, because cooling at high temperatures is faster in a sinusoidal model than that in a linear model. As a result, if the natural situation

shows some extent of similarity with a sinusoidal cooling model, the average cooling rate might be slightly larger than those determined is this study using a constant cooling rate. Nevertheless, the potential uncertainty derived from a nonlinear cooling path would not undermine the conclusion indicating a rapid cooling rate.

(3) There is potential uncertainty in applying the experimentally determined CaAl-NaSi interdiffusion coefficients. Firstly, plagioclase composition has a negative effect on diffusivity (Fig. S5; refs. [19,25,26]). In this case, the plagioclase compositions are around An_{50}-An_{70}, whereas the best calibrated diffusion coefficients were performed for plagioclase compositions within An_{70}-An_{90}. Therefore, using these diffusion coefficients, the modelled cooling rates should be lower limits compared to the real values. Secondly, water activity also exerts an important effect on the diffusivity (Fig. S5; refs. [19,23,25-27]). Because amphibole is a common phase in the altered sheeted dikes, we suggest that the water activity is high (maybe near 1) in the system during the hydrothermal cooling process, and thus diffusivities determined only at hydrous should be applied. However, the water activity in this case is difficult to estimate quantitatively. Two independent diffusion experiments of Liu and Yund (1992)[19] and Baschek and Johannes (1995)[27] give very different results (Fig. S7), showing that the diffusion coefficients determined by Baschek and Johannes (1995)[27] at a water undersaturated condition (X_{H2O}=0.5, mixed with CO_2) are over one order of magnitude higher than those determined by Liu and Yund (1992)[19] at hydrous condition (pure water). In this study, we chose the diffusion coefficients for a hydrous system and plagioclase compositions An_{70}-An_{90} determined by Liu and Yund (1992)[19] for our modelling, which is the lower limit of available data for CaAl-NaSi interdiffusion at hydrous conditions. As a result, our modelled cooling rate might be a lower limit value considering the potential uncertainty in the diffusion rate, assuming that the water activity during the hydrothermal cooling process is high and near 1. If the real water activity is much lower than 1, the diffusivity is lower and, hence, the derived cooling rate is also lower. Another argument for high water activities during the contact metamorphic overprint is given by the fact that the sheeted dikes above the axial melt lens experienced hydrothermal alteration before the metamorphic imprint[6,12], providing water-rich fluids deliberated by the breakdown of hydrous minerals during the prograde metamorphism.

(4) The uncertainty in temperature estimation for peak thermal overprint has an important potential effect on the cooling rate modelling. In this study, we estimated the temperatures of thermal overprint by applying the two-pyroxene geothermometer[28] and used the maximum values as peak temperatures for each sample. In the calculation, the pressures are set as 0.5 kbar since our samples were collected from an intact ocean crust at depths of about 1500 mbsf, and thus potential uncertainty in the thermometer from pressure is negligible. However, the uncertainty in using the two-pyroxene geothermometer has not been well constrained. Here we assume a nominal uncertainty of ±15 °C for the temperature estimation

of peak thermal overprint, which may propagate into cooling rate modeling and lead to relative errors within 30-70% for the diffusion profiles we have measured. An example is shown in Fig. S12.

In summary, evaluations on the potential errors in spatial resolution of the electron microprobe, in cooling paths, in experimentally determined diffusion coefficients, and in temperature estimation for peak thermal overprint indicate that our modelled cooling rates using the assumptions are reasonable within relative errors of a factor of 2. The largest uncertainty comes from the quality of the diffusion data for plagioclase and improved determination of chemical diffusivity in function of An content, water activity and temperature is required to make the estimation of cooling rates more robust.

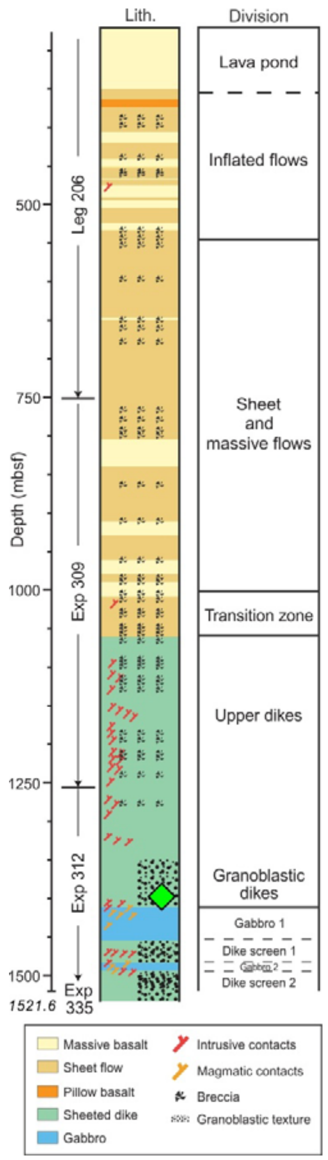

Figure S1: Simplified lithology stratigraphy of IODP Hole 1256D, showing the dominant rock types[2,3]. Samples used for this study are pyroxene hornfelses from the horizon named “granoblastic dikes” by refs. 2-3. The green diamond denotes the positions of samples 203R-1-10_14 and 205R-1-10_14, whereas the positions of R12-B and R12-S were both collected from junk basket, and their depths are unable to constrain precisely. For details see ref. 3.

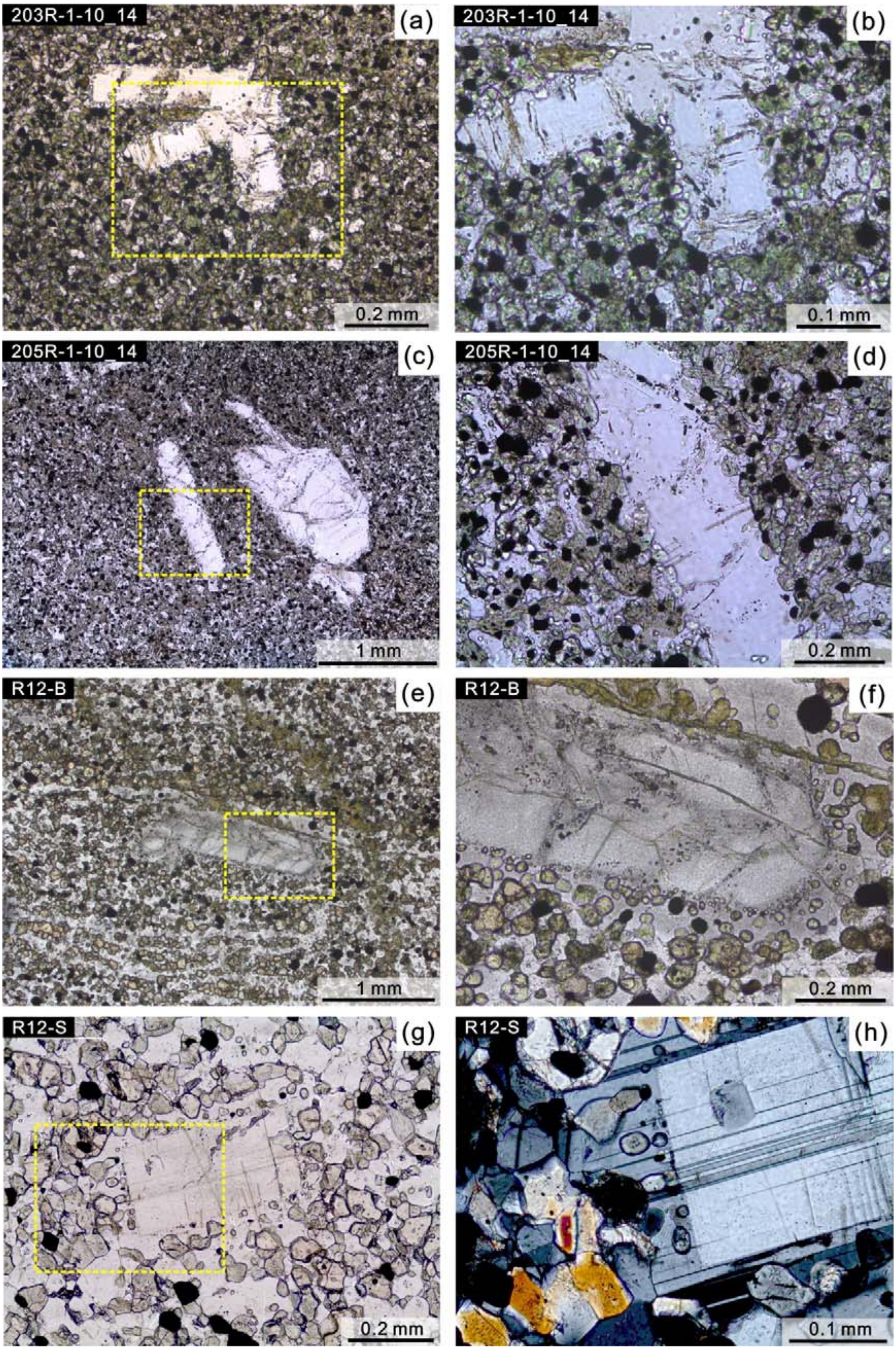
203R-1-10_14
(a)
0.2 mm
203R-1-10_14
(b)
0.1 mm
205R-1-10_14
(c)
1 mm
205R-1-10_14
(d)
0.2 mm
R12-B
(e)
1 mm
R12-B
(f)
0.2 mm
R12-S
(g)
0.2 mm
R12-S
(h)
0.1 mm

Figure S2: Thin section photomicrographs of selected pyroxene hornfelses from “granoblastic dike” horizon. **203R-1-10_14**: A cluster of three euhedral Plg phenocrysts which survived in a granoblastic matrix composed of granular Plg, Cpx, Opx and Ox (a). As shown in the zoom-in view (b), the outer part of the Plg phenocrysts encloses numerous tiny microgranular Cpx, which can also be observed in some matrix granular Plg. **205R-1-10_14**: Euhedral igneous Plg phenocrysts are surrounded by a fine-grained granoblastic matrix (c). The overgrowth upon the Plg phenocrysts is very thin and contains no apparent microgranular inclusion (d). **R12-B**: In the center of (e) is a euhedral Plg phenocryst (>1 mm in length) showing a distinctive core-rim texture, which is interpreted as relic magmatic Plg (core) with granoblastic overgrowth (rim). In the zoom-in view (f), microgranular grains are observable adjacent to the core-rim boundary of the Plg phenocryst. At the bottom of (e), granular Opx (pink in color) are arranged in near-horizontal vein-like textures. Local greenish Amp can be observed as veins and is interpreted to be formed by later hydrothermal alteration. **R12-S**: An euhedral platy Plg phenocryst is surrounded by granoblastic matrix of granular Cpx, Opx, Plg and Ox (g). The photomicrograph under cross-polarized light (h) shows that the igneous Plg phenocryst (bright in interference color) has different extinction position from the overgrowth (dark in interference color), and the latter encloses tiny granular Cpx, Opx and Ox.

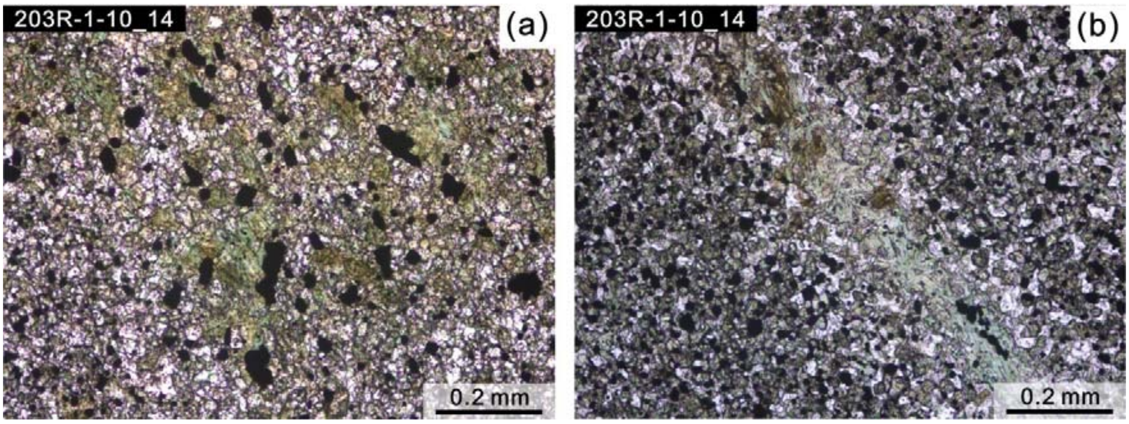

Figure S3: Thin section photomicrographs of pyroxene hornfelses sample 203R-1-10_14 showing the record of a hydrothermal imprint after the peak of the granoblastic recrystallization. (a) Sporadic amphibole grains within the granoblastic domain (coexisting with large secondary Fe-Ti oxides) implies a hydrothermal alteration mainly of Cpx. (b) A veinlet cutting the granoblastic domain, which is mainly composed of bluish chlorite.

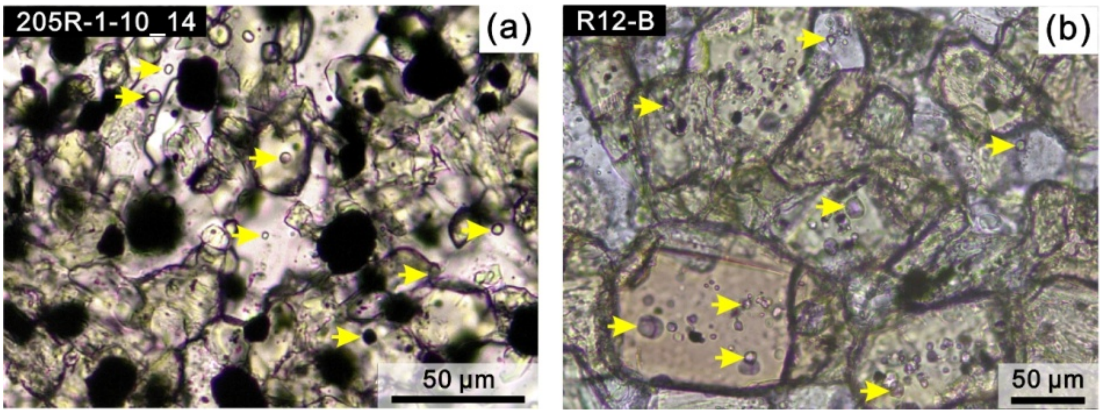

Figure S4: Thin section photomicrographs showing microgranular inclusions. The microgranular inclusions (indicated with arrows), characterized by rounded shapes and tiny sizes ranging mainly within ca. 1-10 μm, are ubiquitous in the investigated hornfelses. Microgranular inclusions are mainly Plg, Cpx, Opx, Mgt and Ilm, and rarely sulfides, analogous to that of rock matrix and thus interpreted as inherited nuclei from an earlier stage of granoblastic overprint. Overview of thin sections indicates that the microgranular inclusions in R12-B seem more abundant than those in 205R-1-10_14.

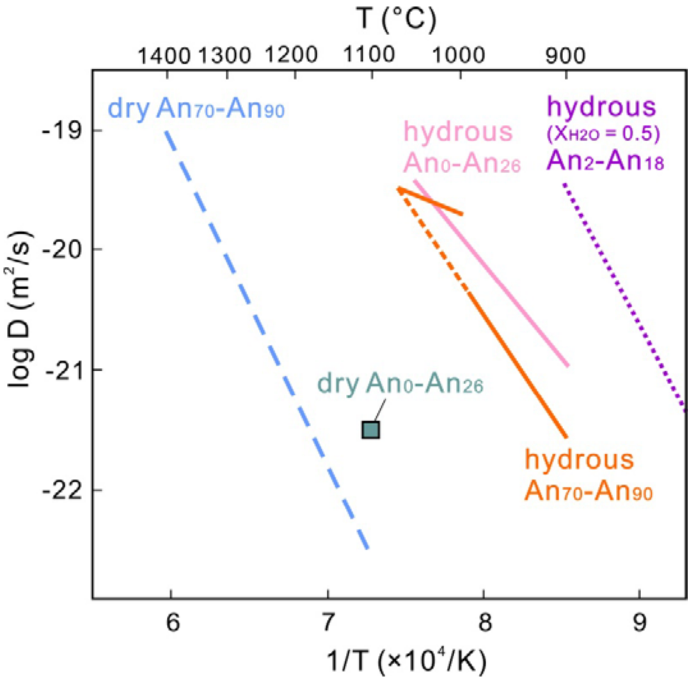

Figure S5: Summary of literature data of CaAl-NaSi interdiffusion. Data sources: dry An_{70}-An_{90}, ref. [25]; dry An_{0}-An_{26}, ref. [26]; hydrous An_{0}-An_{26} and An_{70}-An_{90}, ref. [19]; hydrous (X_{H2O} = 0.5) An_{2}-An_{18}, ref. [27]. The dashed line for hydrous An_{70}-An_{90} is extrapolated from lower-temperature data, and the equation of this line is used for modeling in this study.

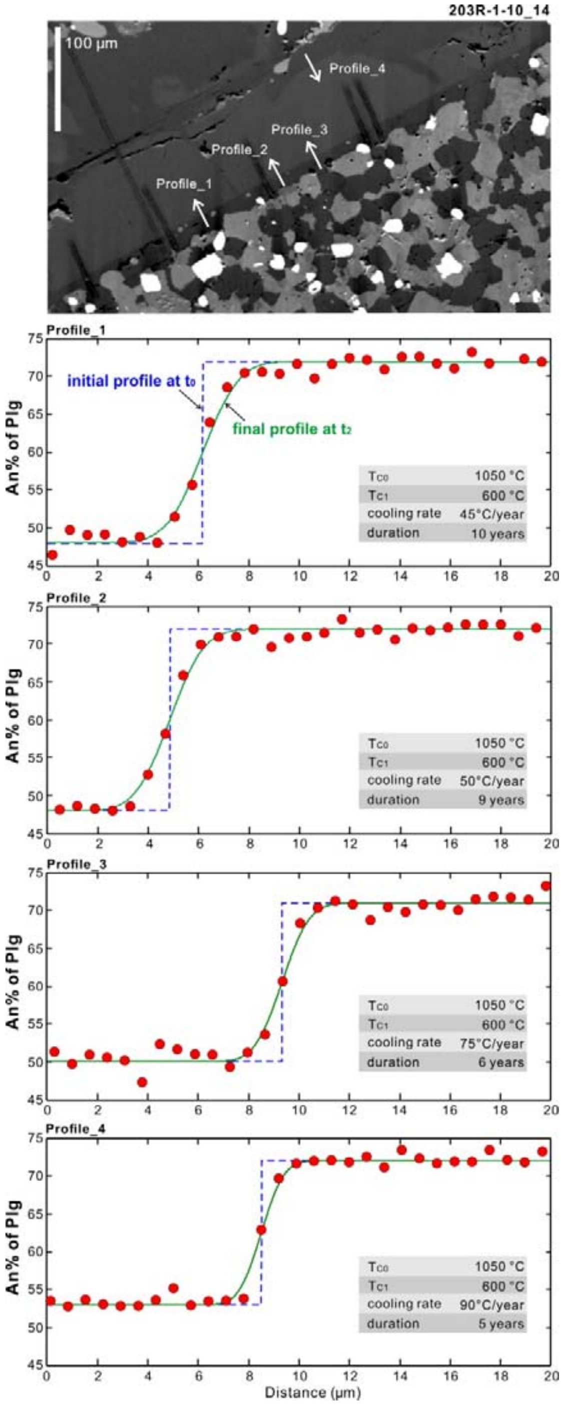

Figure S6: Intra-plagioclase zoning patterns and diffusion modelling for sample 203R-1-10_14.

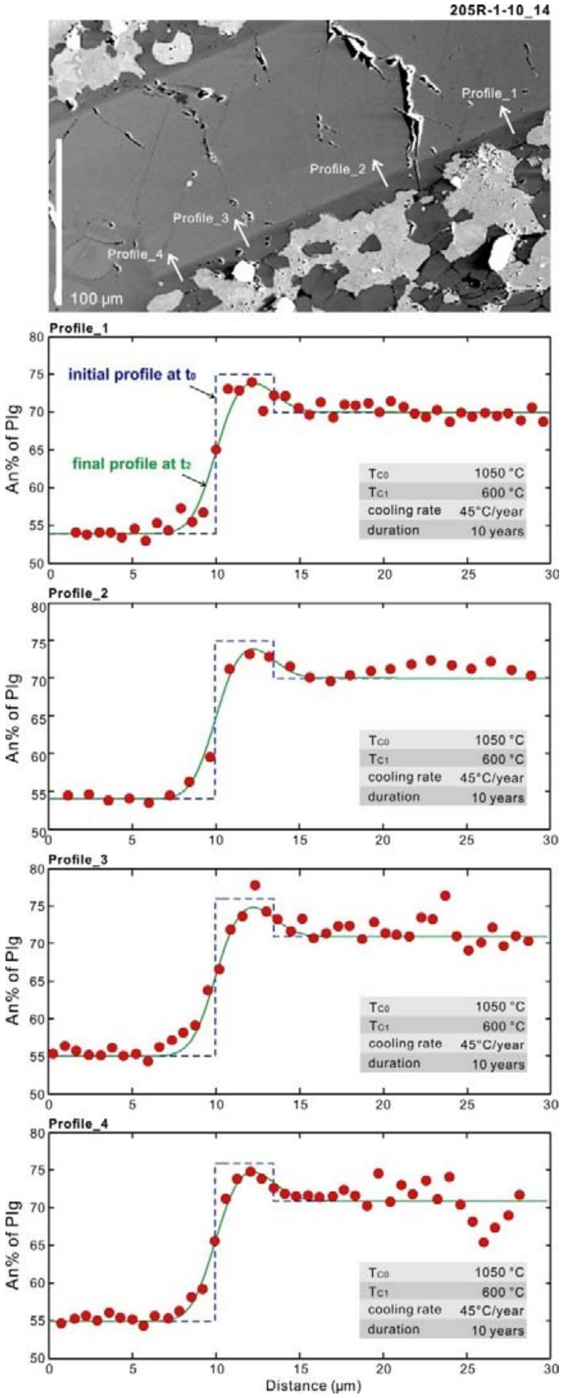

296
297 **Figure S7:** Intra-plagioclase zoning patterns and diffusion modelling for sample 205R-1-10_14.
298

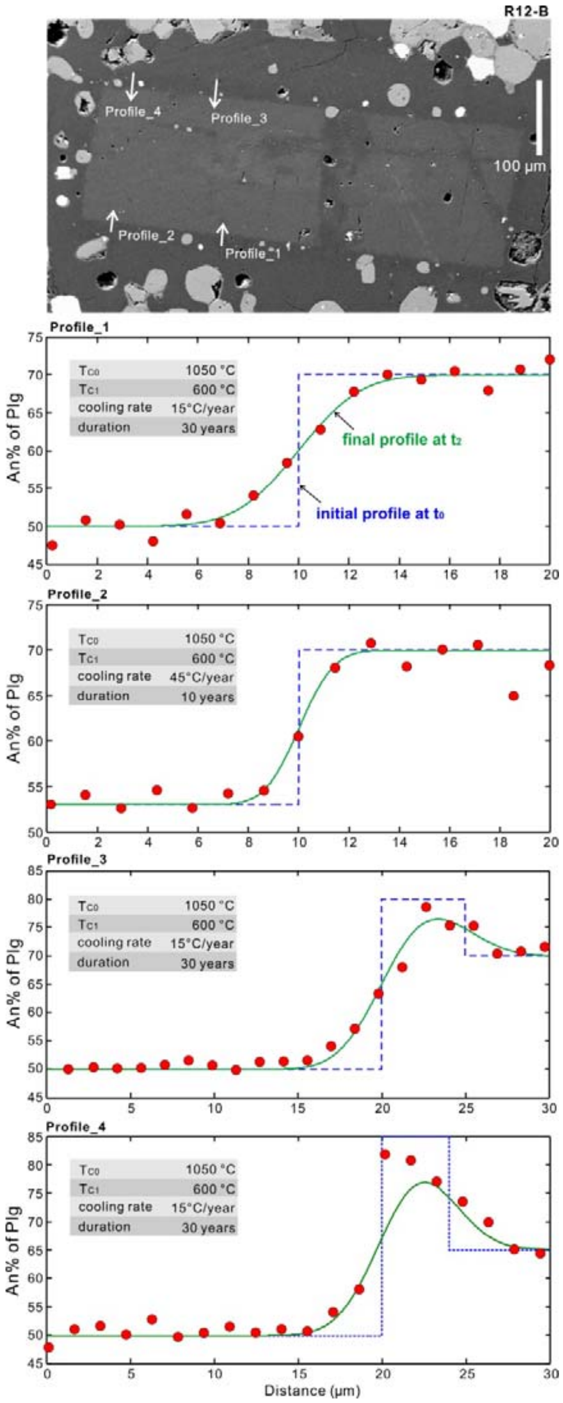

Figure S8: Intra-plagioclase zoning patterns and diffusion modelling for sample R12-B.

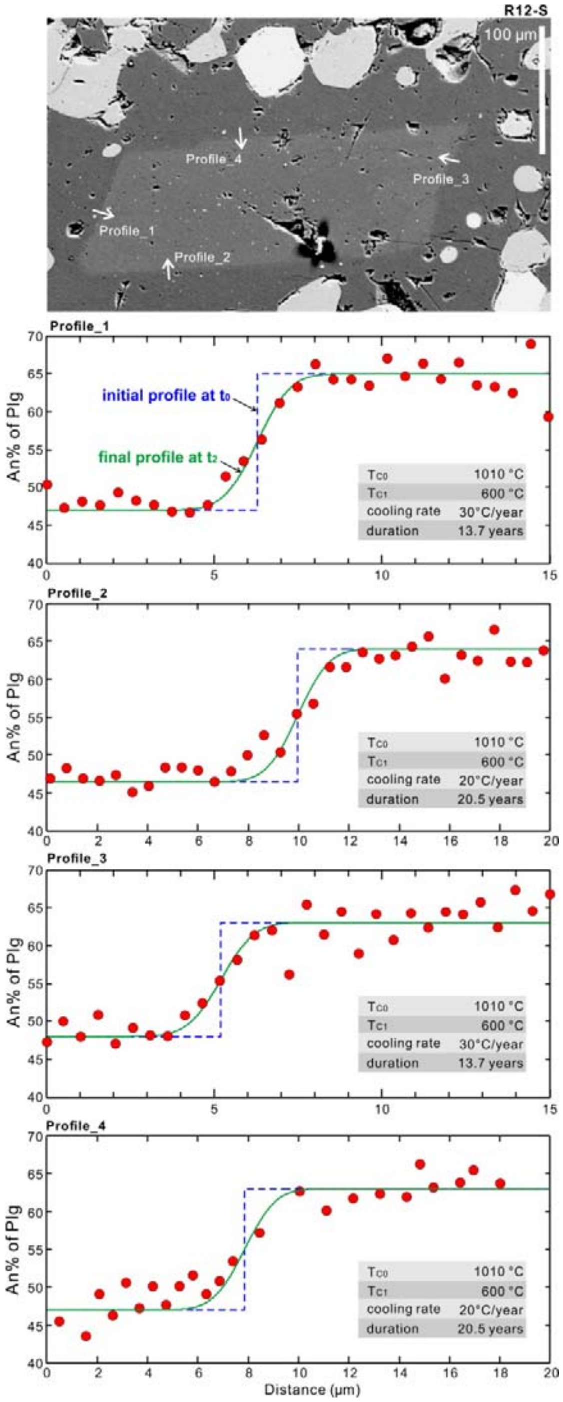

Figure S9: Intra-plagioclase zoning patterns and diffusion modelling for sample R12-S.

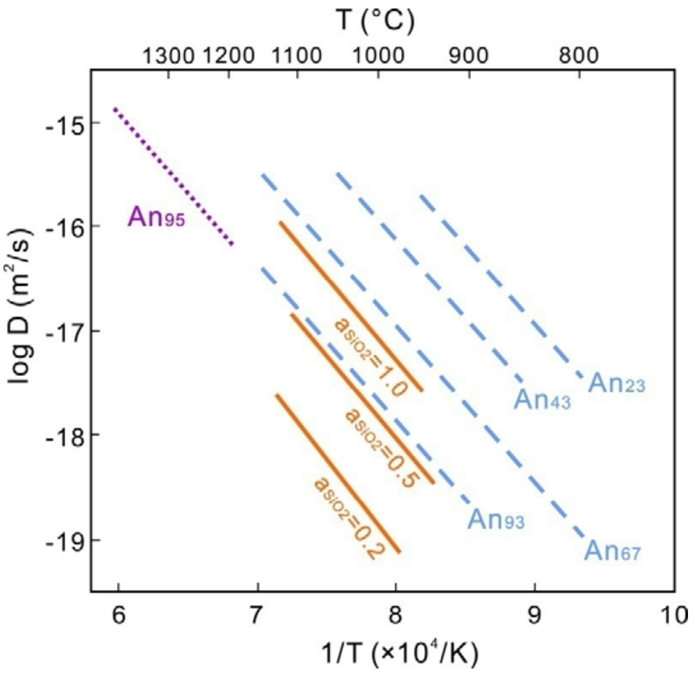

Figure S10: Summary of literature data of Mg diffusion in plagioclase. Data sources: An_{95} (dotted line), ref. [20]; An_{23}, An_{43}, An_{67} and An_{93} (dashed lines), ref. [21]; a_{SiO2}=1.0, a_{SiO2}=0.5 and a_{SiO2}=0.2 (solid lines, An = 60%), calculated according to ref. [22].

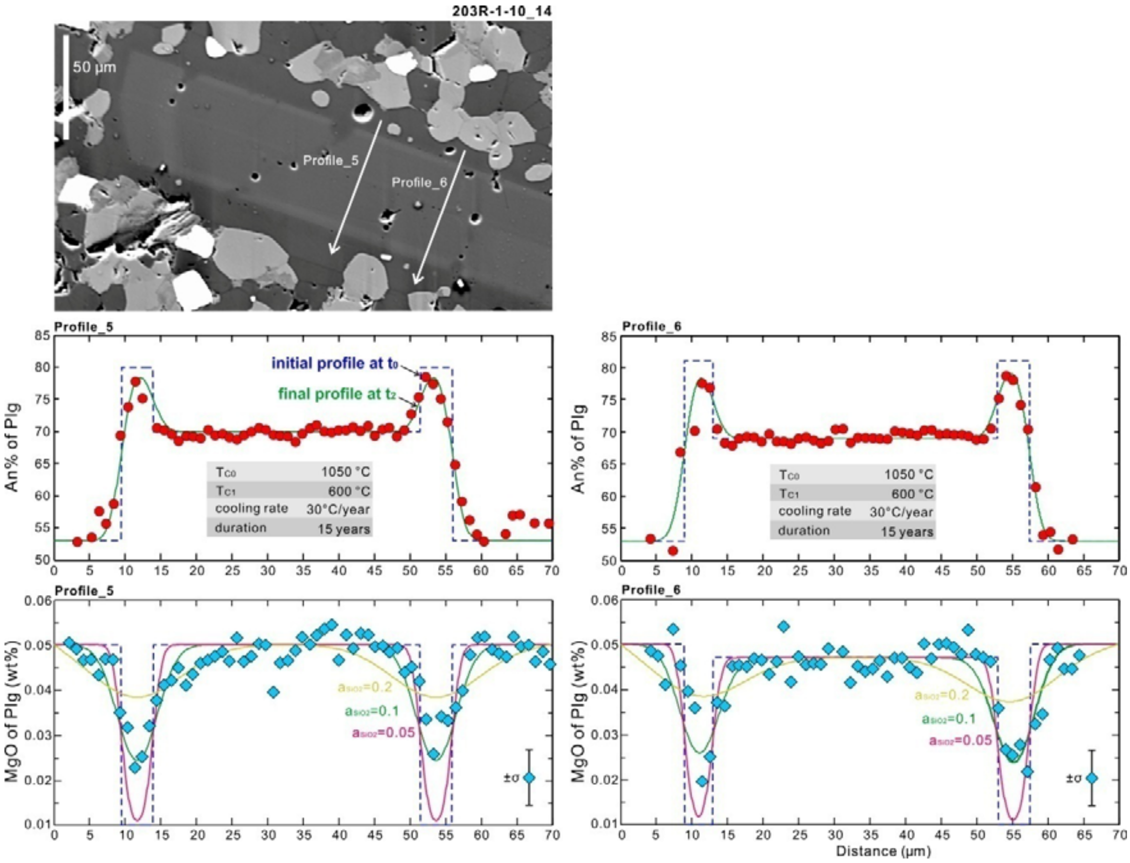

Figure S11: concentration-distance profiles and modelling results for intra-plagioclase CaAl-NaSi and Mg diffusion for sample 203R-1-10_14. The modelling for Mg diffusion indicates low SiO_2 activities (a_{SiO2}) far below 1.

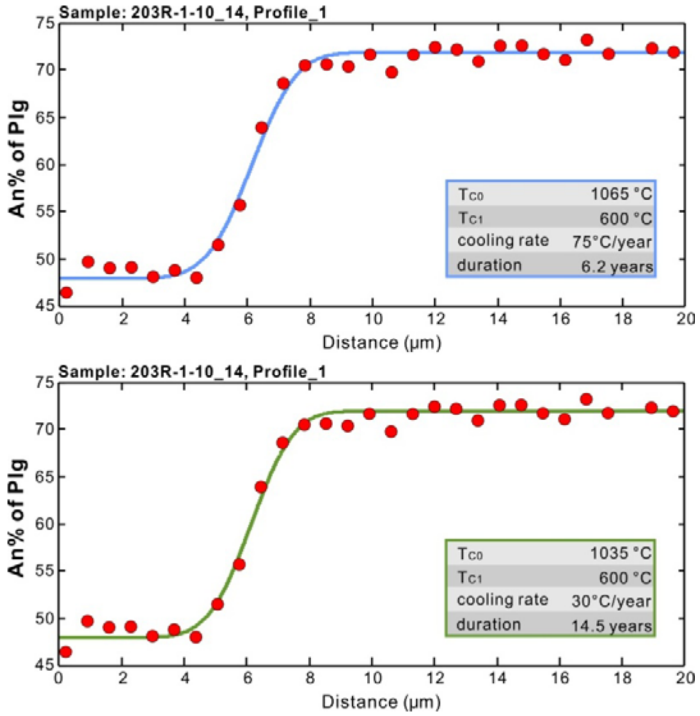

Figure S12: An example showing error propagation from the uncertainty in temperature estimation of peak thermal overprint into the uncertainty in cooling rate modelling. For temperature estimation applying two-pyroxene thermometer, a nominal uncertainty of ±15 °C was considered. In the example of profile_1 of sample 203R-1-10_14 for which a peak temperature of 1050 °C gives a cooling rate of 45 °C/year (see Fig. S6), a higher peak temperature of 1065 °C would give a cooling rate of 75 °C/year (relative error is 67%) and a lower peak temperature of 1035 °C would give a cooing rate of 30 °C/year (relative error is 50%).

References

1. Wilson, D. S. Fastest known spreading on the Miocene Cocos-Pacific Plate Boundary. *Geophys. Res. Lett.* **23,** 3003-3006 (1996).
2. Teagle, D. A. H., Alt, J. C., Umino, S., S. Miyashita, Banerjee, N. R., Wilson, D. S.*, et al.* Proceedings of the Integrated Ocean Drilling Program, vol. 309/312. Ocean Drill. Program (2006).
3. Teagle, D. A. H., Ildefonse, B., Blum, P. & Scientists, t. E. Proceedings of the Integrated Ocean Drilling Program, vol. 335. Tokyo: Integrated Ocean Drilling Program Management International, Inc. (2012).
4. Wilson, D. S., Teagle, D. A. H., Alt, J. C., Banerjee, N. R., Umino, S., Miyashita, S.*, et al.* Drilling to Gabbro in Intact Ocean Crust. *Science* **312,** 1016-1020 (2006).
5. Koepke, J., France, L., Müller, T., Faure, F., Goetze, N., Dziony, W.*, et al.* Gabbros from IODP Site 1256, equatorial Pacific: Insight into axial magma chamber processes at fast spreading ocean ridges. *Geochem. Geophys. Geosyst.* **12,** Q09014 (2011).
6. Koepke, J., Christie, D. M., Dziony, W., Holtz, F., Lattard, D., Maclennan, J.*, et al.* Petrography of the dike-gabbro transition at IODP Site 1256 (equatorial Pacific): The evolution of the granoblastic dikes. *Geochem. Geophys. Geosyst.* **9,** Q07O09 (2008).
7. France, L., Ildefonse, B. & Koepke, J. Interactions between magma and hydrothermal system in Oman ophiolite and in IODP Hole 1256D: Fossilization of a dynamic melt lens at fast spreading ridges. *Geochem. Geophys. Geosyst.* **10,** Q10O19 (2009).
8. Fischer, L. A., Erdmann, M., France, L., Deloule, E., Zhang, C. & Koepke, J. Generation of felsic melts within fast-spreading oceanic crust: Experimental partial melting of hydrothermally altered sheeted dike. *AGU*. San Francisco, Calif. (2013).
9. Gillis, K. M. & Coogan, L. A. Anatectic Migmatites from the Roof of an Ocean Ridge Magma Chamber. *J. Petrol.* **43,** 2075-2095 (2002).
10. Beard, J. S. Chapter 17: Partial melting of metabasites in the contact aureoles of gabbroic plutons in the Smartville Complex, Sierra Nevada, California. *Geol. Soc. Am. Mem.* **174,** 303-314 (1990).
11. Boudier, F., Godard, M. & Armbruster, C. Significance of gabbronorite occurrence in the crustal section of the Semail ophiolite. *Mar. Geophys. Res.* **21,** 307-326 (2000).
12. Alt, J. C., Laverne, C., Coggon, R. M., Teagle, D. A. H., Banerjee, N. R., Morgan, S.*, et al.* Subsurface structure of a submarine hydrothermal system in ocean crust formed at the East Pacific Rise, ODP/IODP Site 1256. *Geochem. Geophys. Geosyst.* **11,** Q10010 (2010).
13. Johannes, W., Koepke, J. & Behrens, H. Partial Melting Reactions of Plagioclases and Plagioclase-Bearing Systems. In: Parsons I (ed). *Feldspars and their Reactions*, vol. 421. Springer Netherlands, pp 161-194 (1994).
14. Costa, F., Dohmen, R. & Chakraborty, S. Time scales of magmatic processes from modeling the zoning patterns of crystals. *Reviews in Mineralogy and Geochemistry* **69,** 545 (2008).
15. Kahl, M., Chakraborty, S., Costa, F. & Pompilio, M. Dynamic plumbing system beneath volcanoes revealed by kinetic modeling, and the connection to monitoring data: An example from Mt. Etna. *Earth Planet. Sci. Lett.* **308,** 11-22 (2011).
16. Maclennan, J., Hulme, T. & Singh, S. C. Cooling of the lower oceanic crust. *Geology* **33,** 357-366 (2005).
17. Crank, J. *The mathematics of diffusion*. Clarendon Press: Oxford (1975).
18. Cherniak, D. J. Cation Diffusion in Feldspars. *Rev. Mineral. Geochem.* **72,** 691-733 (2010).

19. Liu, M. & Yund, R. A. NaSi-CaAl interdiffusion in plagioclase. *Am. Mineral.* **77,** 275-283 (1992).
20. LaTourrette, T. & Wasserburg, G. J. Mg diffusion in anorthite: implications for the formation of early solar system planetesimals. *Earth Planet. Sci. Lett.* **158,** 91-108 (1998).
21. Van Orman, J. A., Cherniak, D. J. & Kita, N. T. Magnesium diffusion in plagioclase: Dependence on composition, and implications for thermal resetting of the 26Al–26Mg early solar system chronometer. *Earth Planet. Sci. Lett.* **385,** 79-88 (2014).
22. Faak, K., Chakraborty, S. & Coogan, L. A. Mg in plagioclase: Experimental calibration of a new geothermometer and diffusion coefficients. *Geochim. Cosmochim. Acta.* **123,** 195-217 (2013).
23. Behrens, H., Johannes, W. & Schmalzried, H. On the mechanisms of cation diffusion processes in ternary feldspars. *Phys. Chem. Miner.* **17,** 62-78 (1990).
24. Rannou, E., Caroff, M. & Cordier, C. A geochemical approach to model periodically replenished magma chambers: Does oscillatory supply account for the magmatic evolution of EPR 17–19°S? *Geochim. Cosmochim. Acta.* **70,** 4783-4796 (2006).
25. Grove, T. L., Baker, M. B. & Kinzler, R. J. Coupled CaAl-NaSi diffusion in plagioclase feldspar: Experiments and applications to cooling rate speedometry. *Geochim. Cosmochim. Acta.* **48,** 2113-2121 (1984).
26. Yund, R. A. Interdiffusion of NaSi—CaAl in peristerite. *Phys. Chem. Miner.* **13,** 11-16 (1986).
27. Baschek, G. & Johannes, W. The estimation of NaSi-CaAl interdiffusion rates in peristerite by homogenization experiments. *Eur. J. Mineral.* **7,** 295-307 (1995).
28. Andersen, D., Lindsley, D. & Davidson, P. QUILF: A pascal program to assess equilibria among Fe-Mg-Mn-Ti oxides, pyroxenes, olivine, and quartz. *Comp. Geosci.* **19,** 1333-1350 (1993).
